# Multi-Order Asymmetric Acoustic Metamaterials with Broad Bandgaps at Subwavelength Scales

**DOI:** 10.3390/ma16247587

**Published:** 2023-12-10

**Authors:** Xiaopeng Wang, Wenjiong Chen, Sheng Li

**Affiliations:** State Key Laboratory of Structural Analysis, Optimization and CAE Software for Industrial Equipment, Dalian University of Technology, Dalian 116024, China

**Keywords:** broad bandgap, multi-order, asymmetric, acoustic metamaterials

## Abstract

Noise manipulation at the subwavelength scale remains a challenging problem. To obtain better broadband sound isolation within the subwavelength range, a class of asymmetric acoustic metamaterials (AAMs) based on rotation is proposed, and this class of AAMs can further improve subwavelength sound isolation performance by introducing multi-orders. The influences of changing the alternate propagation length of the coiled channel and the square cavity in the unit cell on the band frequency distribution and the omnidirectional band structure were investigated. The effective parameters are calculated with the S-parameter retrieval method, and the generation and change mechanisms of the bandgaps were elucidated. The calculation of sound transmission characteristics showed that, in the asymmetric mode, the overall sound isolation performance of the structure was greatly improved, and the relative bandwidth expanded as the alternate propagation length of the coiled channel and square cavity increased. The omnidirectional bandgaps from the first-order to the third-order AAMs occupied 63.6%, 75.96%, and 76.84% of the subwavelength range, respectively. In particular, the first bandgap moves to the low frequency and becomes wider. Both the experimental results and numerical analyses consistently showed that disrupting structural symmetry enhances acoustic metamaterials for superior broadband sound isolation, inspiring broader applications for asymmetry in this field.

## 1. Introduction

Acoustic metamaterials are artificial composite materials composed of subwavelength units, which have attracted extensive attention from and research by scientists due to their unusual physical parameters and extraordinary acoustic properties [1]. For example, various physical effects, such as noise control [2], vibration control [3], superfocusing [4], negative refraction [5], and acoustic black holes [6], have very broad application prospects, among which vibration and noise reduction are the most prominent exploration directions [7,8]. However, controlling deep subwavelength scale sound waves and realizing low-frequency broadband sound isolation are still challenging problems at present [9]. This is because reducing the resonant frequency for better low-frequency sound isolation comes at the cost of a narrower resonant bandwidth [10]. Therefore, better design methods and structures are urgently needed for low-frequency broadband noise control.

In terms of broadening the subwavelength sound isolation design, some effective design methods have been developed [11]. Research has found that when a sound wave propagates in the curled/coiled channel inside the material, the effective speed is much lower than the propagation speed in the background medium, and acoustic Mie resonance will be generated under this condition [12,13,14]. Mie resonant acoustic metamaterials have high refractive indexes [15]. This unique acoustic characteristic can effectively prevent the wave propagation of the corresponding frequency and increase the sound isolation bandwidth while meeting low-frequency sound isolation requirements, which opens up a new way through which to conduct sound field manipulation and design new acoustic functional devices. Xiang [16] designed a spatial spiral acoustic metamaterial with quasi-fractal geometric characteristics using a self-similar fractal. The results showed that the maximum percentage of omnidirectional bandgaps was over 33% across the considered spectrum, but each bandgap was discrete and narrow. For ideal bandgap materials, a wide bandgap at a low frequency is usually required because discrete and narrow bandgaps severely limit their potential applications. Liang [17] proposed a vibro-acoustic concurrent topology optimization approach, and an extended multiscale multimaterial interpolation model was developed. However, the final structure of topology optimization will lead to a dramatic increase in the structure’s complexity, and this complexity will not be convenient to manufacture. If the manufacturing constraints are not considered in the optimal design of the structure, to meet the expected requirements, the final structure with topology optimization may have complex perforation patterns and lead to more difficult fabrication [18]. Xu [19] proposed a novel acoustic metamaterial structure composed of resonators and multiple channels to isolate low-frequency broadband noises, which led to the impracticality of the large-scale periodic systems required for low-frequency sound isolation applications in most cases. Moreover, these optimization systems are challenging for practical applications due to their complex structures. Therefore, realizing a practical acoustic metamaterial with a simple structure, broadband attenuation capability, and practicality is still a challenge.

In the study of elastic waves, breaking structural symmetry can reduce the frequency of the bandgap and broaden the bandgap. Tian [20] proposed a design method with which to achieve geometric symmetry breaking by rotating the orientation of the hole, which can effectively create and enlarge the bandgaps. On this basis, Tian [21] proposed a novel type of perforated elastic metamaterial with two bandgap formation mechanisms (i.e., Bragg scattering and local resonance). By introducing four concentric spiral holes into each matrix material with mutually orthogonal rectangular holes, the symmetry of the structure was reduced, resulting in low-frequency and broadband wave attenuation. Therefore, introducing structural asymmetry is an efficient approach through which to create and enlarge the bandgaps in elastic wave metamaterials. However, in the study on controlling subwavelength scale acoustic waves, there are fewer studies on reducing structural symmetry. Shen [22] designed acoustic waveguides with two different winding branch pipes. The coupling effects of the two different winding branch pipes generated resonant vibrations, which constructed an impedance-matching layer and produced sound transmission with a wide band at lower frequencies. Tong [23] reduced structural symmetry by introducing a gradient of channel spacing in the design of gradient space-coiling metamaterials, and this feature opens up possibilities for tuning the equivalent constitutive properties in a more flexible fashion. The bandgap of acoustic metamaterials can be created and enlarged by breaking structural symmetry because destructive interference will change the mode shapes and frequencies of the periodic architected materials, and the Mie resonances state of superposition caused by multipole oscillators will appear [24], thus reconstructing the modal sound pressure distribution and changing the resonant frequency of the unit cells, opening the low-frequency bandgap and expanding the bandgaps. Therefore, it is necessary to conduct further in-depth investigations on the bandgap creation and enlargement mechanisms by breaking the structural symmetry of acoustic metamaterials via basic structure rotation.

In this paper, aiming at the design requirements of subwavelength broadband and the simple unit cell structure of acoustic metamaterials, a class of multi-order asymmetric acoustic metamaterials (AAMs) based on rotation is proposed and demonstrated to improve subwavelength broadband noise suppression performance. In particular, the asymmetric unit cell is directly constructed by rotating the basic structure, and the left–right and up–down symmetry inside of the unit cell are destroyed at the same time to reconstruct the modal sound pressure distribution, change the resonance frequency, and realize subwavelength broadband sound isolation. From the first order to the third order, the overall sound isolation performance of the structure was greatly improved, and the sound isolation bandwidth was expanded from the original narrow frequency to a wider frequency. Firstly, first-order, second-order, and third-order AAMs were constructed by rotating the three basic structures. Secondly, the simplest Brillouin region and the dispersion relation of the asymmetric units were studied, and the effective parameters and acoustic transmission characteristics of the asymmetric units were calculated. Finally, the sound isolation performance of the fabricated model was experimentally tested. The experimental results verified the correctness and effectiveness of the numerical analysis method and calculation results of the AAMs proposed in this study. This study provides a theoretical and technical basis for the development of asymmetric acoustic metamaterials and establishes a promising way to further expand the low-frequency broadband sound isolation applications of acoustic metamaterials.

## 2. Model and Method

### 2.1. Structural Design of Two-Dimensional Asymmetric Acoustic Metamaterials

**Figure 1:**
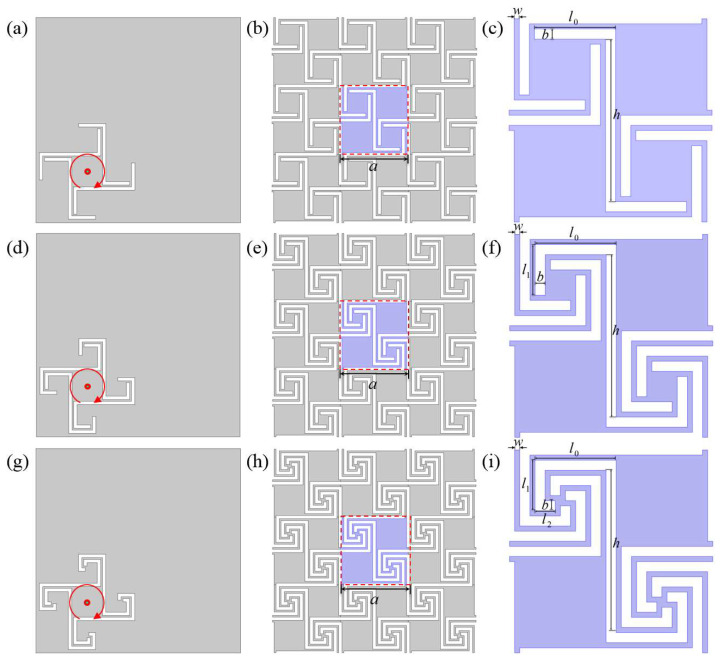
(**a**,**d**,**g**) Construction methods of first-order, second-order, and third-order AAMs. The red arrow is the direction of rotation. (**b**,**e**,**h**) Proposed first-order, second-order, and third-order AAMs with 3 × 3 units. (**c**,**f**,**i**) Key geometric parameters of the first-order, second-order, and third-order unit cells.

Compared with the traditional zigzag channel, self-similar fractal structure, and coiled structure, we introduce the alternating arrangement of the coiled structure and square cavity in the unit design, and the sound wave can propagate along the coiled channel and the square cavity alternately. This alternate arrangement design allows sound waves to enter the square cavity after passing through the coiled channel and produces rich bandgap characteristics. To explore the effect of the transmission path on the acoustic properties of the asymmetric structure, first-order, second-order, and third-order asymmetric units are designed, as shown in Figure 1c,f,i. The sound wave propagates alternately along the coiled channel and the square cavity in the asymmetric structure, and the propagation path is several times longer than the straight path, resulting in the high equivalent refractive index of the asymmetric structure, meaning that the structure has extraordinary acoustic properties.

The two-dimensional units of the first-order, second-order, and third-order AAMs are shown in Figure 1, respectively. In the design process of AAMs, firstly, the three kinds of basic structures composed of solids (white parts in Figure 1a,d,g) are rotated and then arranged periodically in a mutually orthogonal manner (Figure 1b,e,h), and finally, the first-order, second-order, and third-order AAMs formed by the air medium between the solids (Figure 1c,f,i) are obtained. The asymmetric unit cell is directly constructed by rotating the basic structure, which not only breaks the left–right symmetry but also breaks the up–down symmetry so that the unit cell structure becomes a 180° rotational symmetry. By studying the effect of the asymmetry of the units on the dispersion relation, we can guide the design of new configurations characterized by breaking the symmetry.

The lengths of the solids of the three basic structures are defined by *l*_0_, *l*_1_, and *l*_2_, the height is defined by *h*, and the width is defined by *b*. The gap between the solids is the channel for sound wave propagation. In this paper, the width of the sound wave propagation channel of each unit is equal, as defined by *w*. Let the lattice constant *a* = 40 mm, solid width *b* = 2 mm, solid height *h* = 32 mm, *l*_0_ = 16 mm, *l*_1_ = 10 mm, *l*_2_ = 4 mm, and the gap width *w* = 1 mm. The first-order (Figure 1c), second-order (Figure 1f), and third-order (Figure 1i) AAM unit cells in Figure 1 are obtained.

The upper left and lower right corners of the first-order, second-order, and third-order AAM units are all coiled structures, and the propagation path of sound waves in the units can be extended with an increase in the spatial coiled curve. Therefore, using the low effective sound velocity effect in the highly coiled space structure in the acoustic metamaterial to construct a fluid unit with an ultraslow sound velocity can be equivalent to an artificial medium with a higher refractive index. In the proposed AAMs, the sound wave propagates alternately along the coiled channel and the square cavity, so the total propagation length of the sound waves is multiplied. Therefore, the proposed asymmetric unit structure is expected to realize the low frequency of the bandgap, reconstruct the modal sound pressure distribution, change the resonant frequency, and broaden the bandgap.

### 2.2. The Simplest Brillouin Zone of Two-Dimensional Asymmetric Acoustic Metamaterials

To further study the effect of the structural parameters on the acoustic properties and determine the frequency at which the bandgaps occur, the dispersion relation of the asymmetric unit was calculated by the finite element method using the pressure acoustics (acpr) of the commercial software, COMSOL Multiphysics 6.0. The background fluid medium is the air (*ρ*_0_ = 1.21 kg/m^3^, *c*_0_ = 343 m/s) in the coiled channel inside the structure, while the solid material is photosensitive resin (*ρ* = 1130 kg/m^3^, longitudinal elastic wave *c*_p_ = 1545.76 m/s) with high hardness. Therefore, the shear modes within solid structures can be safely ignored during acoustic propagation [25,26]. Specifically, it can be assumed that solids are very hard so that sound waves only propagate in the air channel of two-dimensional acoustic metamaterials. The phonon dispersion relation *ω* = *ω*(*k*) is obtained by solving for the characteristic frequency of the unit cell, where *ω* is the angular frequency and *k* is the wave vector. Without considering viscosity, the governing equations for sound wave propagation in two-dimensional acoustic metamaterials can be written as follows:

(1) $$\nabla \left(\frac{1}{\rho_{0}}\nabla p\right)+\frac{1}{\rho_{0}c_{0}^{2}}\omega^{2}p=0$$

In Equation (1), *ρ*_0_ and *c*_0_ are the air density and sound velocity, and *p* is the sound pressure. The Floquet–Bloch periodic boundary condition is applied to the opposite edges of the unit cell along the *x* and *y* directions, and other boundaries are left free. The sound field *p*_0_ (**r**) must satisfy the following conditions:

(2) $$p_{0}(r+a)=p_{0}(r)e^{-ka}$$

In Equation (2), the wave vector **k** = [*k_x_*, *k_y_*], *k_z_* = 0, and the position vector **r** = [*x*, *y*], *a* is the lattice constant.

**Figure 2:**
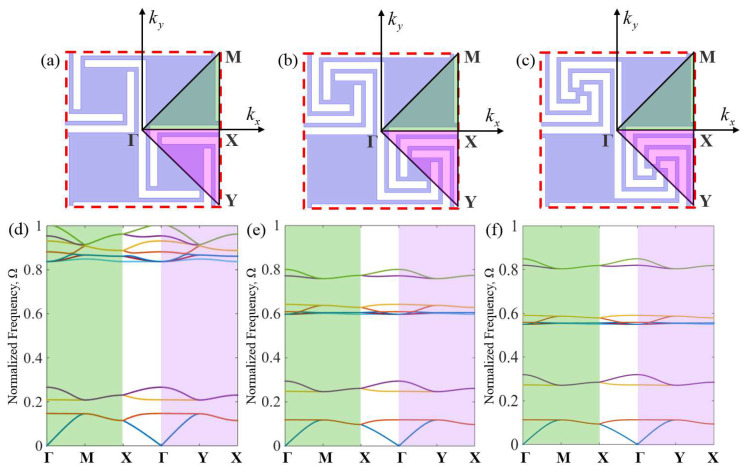
(**a**–**c**) The first Brillouin zone of the square lattice (red dashed box). (**d**–**f**) The dispersion relation curve obtained by scanning the simplest Brillouin zone.

When using the finite element method to solve the dispersion relation, the analysis can be performed for a unit cell, as shown in Figure 2. In the numerical calculation, the model mesh is a free-division triangular mesh. The maximum mesh size is equal to 1/6 of the minimum wavelength, and the mesh size meets the convergence requirement. The wave equation is solved by scanning the wave vector k in the simplest Brillouin region after the unit cell finite element mesh is divided. The characteristic equation in discrete form is as follows:

(3) $$(K-\omega^{2}M)U=0$$

where **U** is the amplitude vector of the sound pressure or displacement of the node, *ω* is the angular frequency of the sound wave or elastic wave propagation, **K** is the effective stiffness matrix, and **M** is the effective mass matrix.

The irreducible Brillouin zone can usually be determined according to the lattice symmetry, but when the shape of the scatterer in the unit cell is irregular, the determination of the irreducible zone also needs to consider the symmetry of the scatterer. If the periodic deviation caused by defects or detuning is small, it is still possible to take a sufficiently large cell, also known as a supercell, assuming the theorem holds and calculates its energy band structure. The computational results still provide a good characterization of the acoustic wave propagation behavior in the structure. The computational results still provide a good characterization of the sound wave propagation behavior in the structure. As shown in Figure 2a–c, because the symmetry of the unit cell structure is broken, the irreducible Brillouin zone is the ΓMXY region, so it is necessary to verify the dispersion curves obtained by wavevector scanning along the ΓMX and ΓMY boundaries for acoustic metamaterials to determine the boundary of the irreducible Brillouin zone. Firstly, this section analyzes the dispersion relationship of the first-order, second-order, and third-order AAMs unit cells in the entire first Brillouin zone shown in Figure 1c,f,i and determines the irreducible Brillouin zone of the AAMs. Secondly, the wave vectors of the first-order, second-order, and third-order unit cells were scanned along the ΓMXΓYX boundary line of the simplest Brillouin zone, and the results are shown in Figure 2d–f. The green and pink shades in the figure represent the dispersion relation obtained by scanning along the ΓXM and ΓYM, respectively. The ordinates in the figure all use the normalized frequency Ω = *fa*/*c*_0_, where *f* is the sound wave frequency corresponding to the Bloch eigenvalue, *c*_0_ is the sound speed of air, and the value *c*_0_ = 343 m/s for this paper. To characterize the acoustic properties of AAMs on the subwavelength scale, the normalized frequency range is restricted to the subwavelength range, that is, 0 < Ω < 1. In Figure 2a–c, the wave vectors in the dispersion curve in the green shade and the pink shade are consistent with the change in frequency. Finally, the dispersion curve obtained by scanning the boundary line along the simplest Brillouin zone is compared with the dispersion surface obtained by scanning the omnidirectional band structure to determine the simplest Brillouin zone of the AAMs. In conclusion, the dispersion diagram of these AAMs can be obtained by scanning along the boundary of the simplest Brillouin zone, and only the dispersion relationship obtained by scanning along the MΓXM boundary is shown below.

### 2.3. Calculation of Effective Parameters

**Figure 3:**
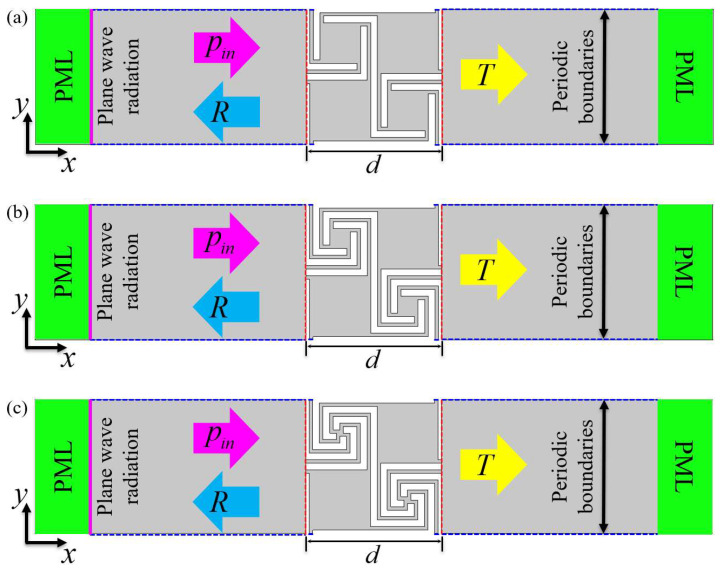
(**a**) Schematic diagram of S-parameter retrieval method from the first-order unit. (**b**) Schematic diagram of S-parameter retrieval method from the second-order unit. (**c**) Schematic diagram of S-parameter retrieval method from the third-order unit.

When sound waves propagate in subwavelength scale acoustic metamaterials, their microstructures cannot be distinguished, which conforms to the dynamic effective medium theory [27]. At this time, the acoustic metamaterial can be regarded as an isotropic homogeneous medium, and its properties can be described by the effective mass density and effective bulk modulus; when the dynamic modulus or density of the material is negative, bandgaps will occur. To deeply understand the mechanism of bandgap generation, it is necessary to determine the resonance mode of Mie resonant acoustic metamaterial in the frequency band where the bandgap appears. Using the “S-parameter retrieval method” to obtain the equivalent parameters of the metamaterial, the resonance mode of the Mie resonant acoustic metamaterial can be determined based on “The monopole resonance can produce a negative bulk modulus, while the dipole resonance can generate a negative mass density [13]”. To verify the negative physical properties of generating bandgaps in AAMs, the effective parameters of AAMs are systematically calculated using the S-parameter retrieval method [27], as shown in Figure 3. A plane wave *p_in_ = p*_0_*e^iωt^* with unit amplitude *p*_0_ = 1 Pa is applied at the left entrance of the waveguide. To simulate a metamaterial with infinite size in the y direction, periodic boundary conditions are applied at the upper and lower ends of the first-order, second-order, and third-order units, and the solid wall is set as a hard boundary condition [28,29]. Perfectly matched layers (PML) are set at the front and back ends of the waveguide to eliminate the influence of reflected waves.

The effective mass density *ρ_eff_* and bulk modulus *B_eff_* of AAMs are given by Equations (4) and (5):

(4) $$\rho_{eff}=\varepsilon \times n$$

(5) $$B_{eff}=\frac{\varepsilon }{n}$$

The effective refractive index *ε* and the effective impedance *n* of AAMs are given by Equations (6) and (7):

(6) $$\varepsilon =\frac{r}{1-2R+R^{2}-T^{2}}$$

(7) $$n=\frac{-i\log x+2\pi m}{kd}$$

where

(8) $$r=\mp \sqrt{\left(R^{2}-T^{2}-1\right)-4T^{2}}$$

(9) $$x=\frac{1-R^{2}+T^{2}+r}{2T}$$

**Figure 4:**
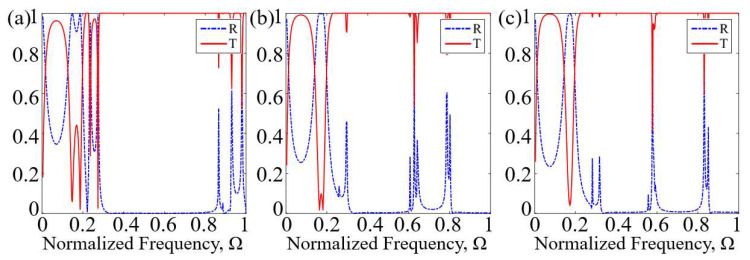
(**a**–**c**) Reflection coefficient *R* and transmission coefficient *T* of the AAMs from the first-order to third-order AAMs.

**Figure 5:**
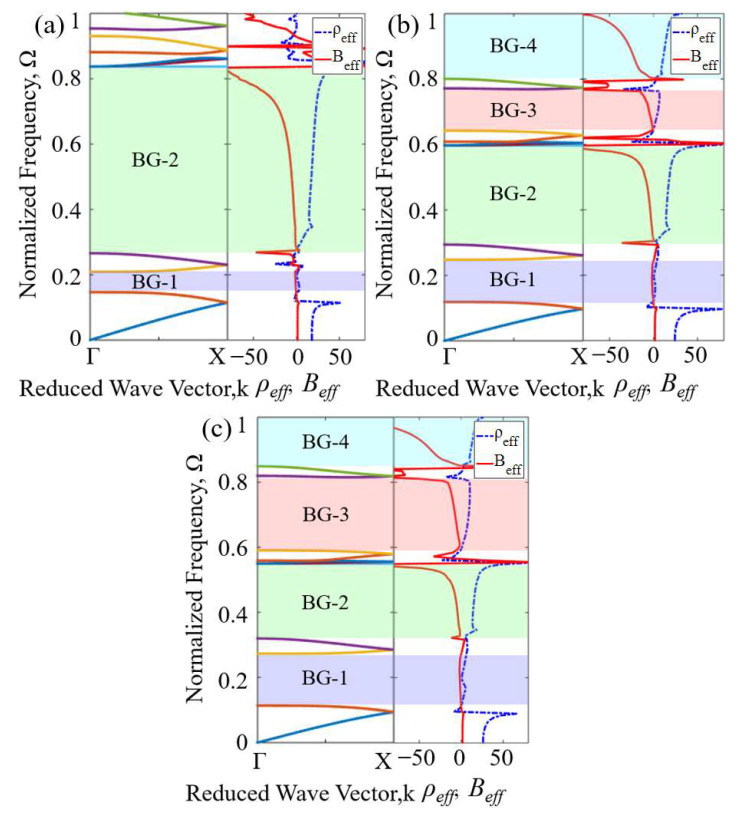
(**a**) The dispersion curves and effective parameter curves of the first-order AAMs along the ΓX direction. (**b**) The dispersion curves and effective parameter curves of the second-order AAMs along the ΓX direction. (**c**) The dispersion curves and effective parameter curves of the third-order AAMs along the ΓX direction. *ρ_eff_* is the blue dashed line, and *B_eff_* is the red solid line.

In the above formulation, *R* is the reflection coefficient of the structure, *T* is the transmission coefficient of the structure, and *k* = ω/*c*_0_ is the acoustic wave number, *d* = *a*. There is no periodic structure in the direction of sound propagation, *m* = 0. The calculation results are shown in Figure 4. The effective parameters of AAMs can be calculated by combining Equations (4)–(9), and the results are shown in Figure 5.

The frequency response curves of the normalized *ρ_eff_* and *B_eff_* in the ΓX direction of the first-order to third-order unit cells are compared with the dispersion relationship in the ΓX direction, as shown in Figure 5a–c. The vertical axis represents the normalized frequency Ω, and the omnidirectional bandgaps are marked with BG-1~BG-4 in the figure. In comparison, it is found that the frequency band where the effective parameter of the material has a negative value was the same as the frequency band where the bandgaps appear in the dispersion relation. In the normalized frequency ranges [0.1470, 0.2097] and [0.2668, 0.8401] of the first-order unit cell in Figure 5a, the effective parameters are negative. In the normalized frequency range [0.1190, 0.2483], [0.2949, 0.5990], [0.6443, 0.7737], and [0.8032, 1] of second-order unit cell in Figure 5b, the effective parameters are negative. In the normalized frequency range [0.1134, 0.2732], [0.3203, 0.5515], [0.5927, 0.8217], and [0.8516, 1] of the third-order unit cell in Figure 5c, the effective parameters are negative. By comparing and analyzing the effective parameters of the first-, second-, and third-order AAM units, the effectiveness of the AAMs for broadband sound isolation in the subwavelength range is demonstrated.

### 2.4. Calculation of Transmission Properties

The calculated results of the band structure show that the AAMs have good low-frequency and broadband properties. Because the width of the winding channel is small, the sound wave propagates in the small-sized coiled channel, and the heat loss and viscous friction loss in the air will cause sound wave attenuation in the channel. The influence of thermal viscous losses on acoustic wave propagation needs to be considered when performing simulation calculations [13,30].

**Figure 6:**
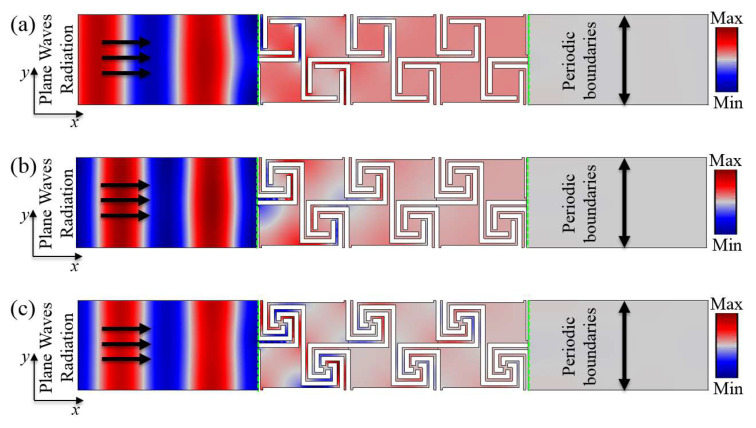
(**a**) Model of wave propagation in the ΓX direction from the first-order AAMs along the ΓX direction. (**b**) Model of wave propagation in the ΓX direction from the second-order AAMs along the ΓX direction. (**c**) Model of wave propagation in the ΓX direction from the third-order AAMs along the ΓX direction.

To further study the acoustic properties of the designed AAMs, thermoviscous acoustics (ta) were used to calculate the sound transmission loss of the first-order, second-order, and third-order AAMs in the ΓX direction, and the dispersion relation was compared with that of the AAMs. The greater the number of structural units, the better the transmission of loss characteristics [28,31,32]. To obtain better sound isolation while avoiding being too large in the transmission direction, we calculated the transmission properties of three identical structural units. The physical field settings are shown in Figure 6. Rectangular waveguides with a length of 80 mm and a width of 40 mm are set at both ends of the acoustic metamaterials. The periodic boundary conditions applied in the y-direction of the waveguide simulate the infinite periodic domain, and perfectly matched layers (PML) are set at the front and back ends of the waveguide to eliminate the influence of the reflected waves. The background pressure field is a plane wave of 1 Pa, which is placed on the left side of the waveguide. Considering that the hardness of the photosensitive resin is much higher than that of the air medium, the hard boundary condition is adopted for the solid boundary. The thermoviscous acoustic module in COMSOL is used to describe the air domain inside the material, and the Helmholtz equation is solved considering finite element discretization. For a given frequency, once the sound pressure field is obtained, the corresponding sound transmission loss (STL) can be obtained from Equation (10):

(10) $$STL\left(\omega \right)=-{10\log}_{10}{\left|\frac{\int_{\Gamma_{t}}p_{t}d\Gamma }{\int_{\Gamma_{i}}p_{b}d\Gamma }\right|}^{2}\left(dB\right)$$

where Γ*_i_* and Γ*_t_* represent the incident edge and the transmission edge, respectively, as shown by the green dotted line in Figure 6. *p_b_* = 1 Pa is the amplitude of the background sound pressure at the incident edge, and *p_t_* is the amplitude of the total sound pressure measured at the transmission edge, which is the sum of the scattered sound pressure and the background sound pressure. However, on the transmission side, the background sound pressure is equal to 0.

**Figure 7:**
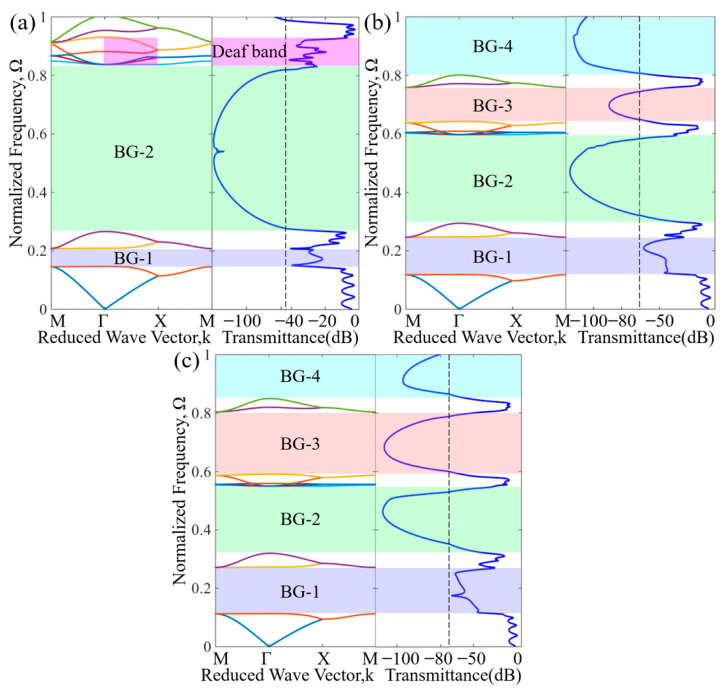
(**a**) Dispersion relation of first-order AAMs and acoustic transmission properties along the ΓX direction. The bandgaps are marked by shaded areas. The dashed black lines denote −45 dB. (**b**) Dispersion relation of the second-order AAMs and acoustic transmission properties along the ΓX direction. The bandgaps are marked by shaded areas. The dashed black lines denote −65 dB. (**c**) Dispersion relation of the third-order AAMs and acoustic transmission properties along the ΓX direction. The bandgaps are marked by shaded areas. The dashed black lines denote −75 dB.

To deeply understand the formation mechanism of the bandgap, the sound wave transmission properties of the omnidirectional bandgap and the deaf band in the subwavelength frequency band were compared. Figure 7a–c shows the dispersion relation and the acoustic transmission properties along the ΓX direction of the first-order to third-order AAMs. By comparing the frequency bands with sound transmission loss in Figure 7a and the frequency bands with negative effective parameters in Figure 5a, it was found that they match well. By comparing the frequency bands with sound transmission loss in Figure 7b and the frequency bands with negative effective parameters in Figure 5b, it is found that they match each other well. By comparing the frequency bands with sound transmission loss in Figure 7c and the frequency bands with negative effective parameters in Figure 5c, it was found that they match well. In the frequency bands with large values of effective parameters, the transmission loss is higher. Among them, the frequency distribution of the omnidirectional bandgaps in the subwavelength range is shown in Table 1.

In Figure 7, except for the area of pink shade markers, the sound transmission loss shows an obvious wave attenuation trend in the omnidirectional bandgaps marked by green and blue shading; that is, the frequency with bandgaps and the frequency with higher transmission loss are very consistent with each other. In addition, there is no directional bandgap in the pink shaded area of the energy band structure diagram, but the acoustic wave in this region is attenuated. This phenomenon is called the deaf band [33]. The resonance modes on the deaf band all show an antisymmetric sound pressure field distribution, that is, when the incident wave propagates in the x direction, it cannot excite the resonance mode, and it appears as a deaf band in the band structure.

The calculation results show that, first, there are new bandgaps in the ΓX direction, and higher transmission loss occurs in the new bandgap. Secondly, with increasing order, the alternate propagation length of the coiled channel and the square cavity increases, leading to an increase in the equivalent transmission path. The cause of this behavior is attributed to the extended transmission paths of the sound waves for higher order AAMs as opposed to the lower order AAMs; thus, the higher order AAMs possess a higher equivalent refractive index. As a result, the frequency of the first bandgap BG-1 gradually decreases, and the bandwidth increases. For the first-order, second-order, and third-order AAMs, the lower bounds of the lowest normalized frequency bandgap are 0.147, 0.119, and 0.113, respectively. In particular, the bandgap of the lowest normalized frequency for the third-order AAMs is [0.1134, 0.2732], where the lower and upper ends of this interval are far lower than 1, thus indicating that the higher-order AAMs are capable of manipulating sound waves with wavelengths far exceeding the size of the unit cell. Finally, from the first-order to the third-order AAMs, the geometric distribution inside the unit cell is increasingly asymmetric. In the asymmetric mode, the generated destructive interference causes the resonance superposition of multipole vibrations, resulting in the proportion of bandgaps in the subwavelength range, and the overall sound isolation performance of the structure is greatly improved. The proportions of the omnidirectional bandgaps for the first-order, second-order, and third-order AAMs are approximately 63.6%, 75.96%, and 76.84% in the subwavelength frequency range, respectively. In particular, the omnidirectional bandgaps percentage of the third-order AAMs is the largest in the subwavelength range, followed by the second-order AAMs and the first-order AAMs. The reason for this phenomenon is that due to the increased geometric asymmetry, high-order AAMs generate stronger resonance at lower frequencies driven by the Mie scattering mechanism [34], thereby broadening the bandgap at lower frequencies. In addition, the proposed AAMs have a broad bandgap and a larger proportion of omnidirectional bandgaps in the subwavelength range compared with the reported fractal-type acoustic metamaterials [16,35] and zigzag-type acoustic metamaterials [36,37]. This shows that we can use AAMs with a structure size that is much smaller than the wavelength in the required frequency range to achieve subwavelength broadband sound isolation. Breaking the symmetry will help to expand the bandwidth and achieve very good low-frequency broadband sound isolation performance, which has broad application prospects in subwavelength sound wave manipulation.

## 3. Experimental Results and Discussion

**Figure 8:**
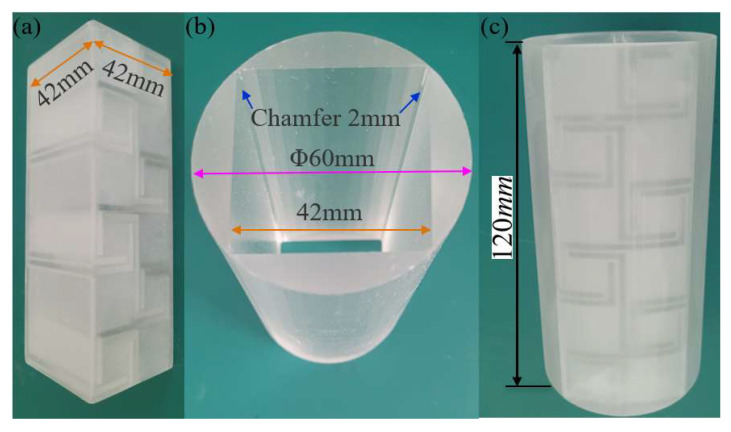
(**a**) The experimental sample. (**b**) The transparent resin mold. (**c**) The combination of the transparent photosensitive resin mold and the test sample.

**Figure 9:**
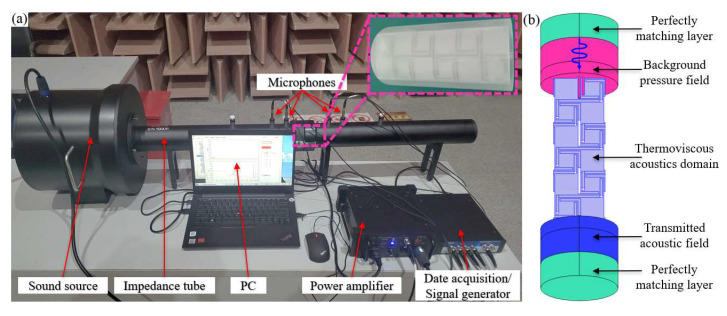
(**a**) 60 mm impedance tube sound isolation test system. (**b**) The 3D finite element model for simulating STL of the sample in impedance tube.

To demonstrate the correctness and effectiveness of the proposed numerical analysis method and calculation results for AAMs in this study, experimental research was conducted. To achieve excellent sound isolation properties, each structural unit needs to be precisely designed because the excellent sound isolation performance of acoustic metamaterials is achieved through the ingenious design of its structure rather than the properties of the material itself. As an artificial microstructure, the acoustic response of metamaterials mainly depends on the size, shape, and arrangement of the micro-resonance units. Therefore, we only fabricated the first-order AAM test sample with photosensitive resin 3D printing. The unit geometric parameters of the sample used in the test were *a* = 40 mm, *b* = 2 mm, *w* = 1 mm, *h* = 32 mm, *l*_0_ = 16 mm, and a cuboid structure with length × width × height = 40 × 40 × 120 mm was adopted, as shown in Figure 8. For the correctness of the simulation results, the material parameters and geometric parameters used in the simulation calculation are consistent with the experimental samples, the boundary conditions are consistent with the experimental environment, and the heat loss and viscous loss in the coiled channel are comprehensively considered. Considering that the stiffness of the photosensitive polymer resin structure is much higher than that of the air medium, a hard sound field boundary condition is applied at the boundary of the model, as shown in Figure 9b. The perfectly matched layers (PML) in the figure represent an infinite air region that absorbs all outgoing and reflected waves. The background sound pressure field is a plane wave sound field incident to the structure surface along the arrow direction.

As shown in Figure 9, the experimental setup consisted of a computer with test software installed, a 60 mm impedance tube (BSWA, Beijing, China, SW9115) with a frequency range of 100 Hz to 2500 Hz, a 300 W power amplifier (BSWA, Beijing, China, PA300), and a 60 mm impedance tube system with a frequency range of 100 Hz to 2500 Hz. 4 channel signal collector (BSWA, Beijing, China, MC3242A) and several connections, etc. The built-in signal generator of the test software generates a broadband Gaussian white noise signal, and the speaker in the impedance tube is driven by a power amplifier to emit white noise to excite the sample placed inside the impedance tube, as shown in Figure 9a. To prevent sound leakage, vaseline is used to seal the gap between the test sample and the inner wall of the impedance tube.

**Figure 10:**
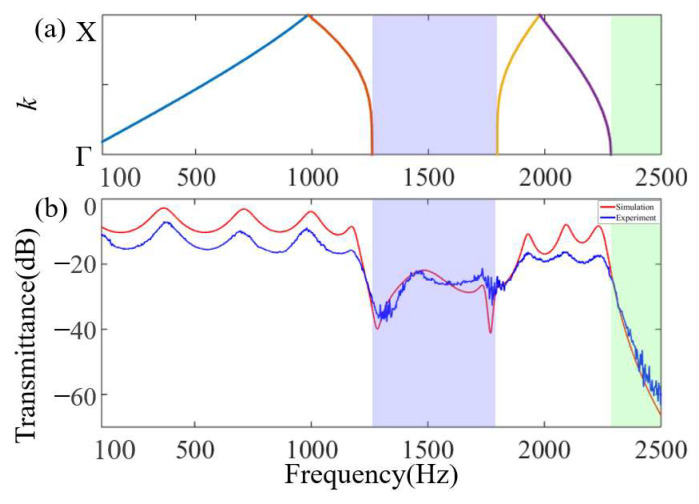
(**a**) The dispersion relationship of first-order AAMs in the ΓX direction. (**b**) The sound transmission loss of the simulation and experiment; the red solid line represents the simulation calculation, and the blue solid line represents the experimental test.

The numerical simulation results and experimental test results are shown in Figure 10. This verifies that the bandgaps of the proposed AAMs are real and can achieve a good broadband sound isolation effect at the subwavelength scale. In Figure 10b, the frequency band (1257–1795 Hz) covered by the blue shadow represents the BG-1 bandgap frequency position, and the amplitude of sound attenuation reaches 40 dB. The frequency band (2284–2500 Hz) covered by the green shadow represents the BG-2 bandgap frequency position with an acoustic attenuation amplitude of up to 60 dB. In the ranges of 100–1210 Hz and 1800–2260 Hz, there is a certain amplitude difference between the simulation results and the experimental results, but the peak frequencies are almost the same. The main source of the amplitude error is the neglect of solid structure damping in the numerical model, which will reduce the vibration energy at the resonance frequency, causing the peak value of the resonance frequency in the experimental results to be low. This is because the thermal viscosity of the narrow coiled channels has a greater impact on wave transmission, which leads to a large difference. In addition, there are some manufacturing errors in the sample manufacturing, and the sample surface is not absolutely smooth relative to the simulated surface, which will lead to an additional increase in the actual sound energy viscosity loss. In the frequency range marked by the blue shade, there are attenuations and peak offsets in the experiment, and the maximum differences between the amplitude and peak are 5 dB and 34 Hz, respectively. The attenuation in the peak value and the shift of the peak frequency can be attributed to the reduction in the wave velocity due to dissipation in the waveguide channel, resulting in energy loss and frequency shift. In addition, the friction between the air vibration and the wall of the structure causes some energy loss.

## 4. Conclusions

In this research work, to obtain a better low-frequency broadband sound isolation effect in the subwavelength range, a class of multi-order AAMs generated by a rotating basic structure is designed to manipulate sound transmission on the subwavelength scale. The research shows that in the asymmetric mode, the overall sound isolation performance of the structure is greatly improved, and the relative bandwidth increases with an increase in the alternate propagation length of the coiled channel and the square cavity. The omnidirectional bandgaps from the first-order to the third-order AAMs occupy 63.6%, 75.96%, and 76.84% of the subwavelength range, respectively. In particular, the first bandgap moves to the low frequency and becomes wider, and higher-order AAMs show a larger proportion of bandgaps, which proves that the proposed multi-order AAMs can achieve subwavelength broadband sound isolation. In this research work, the wave vector and frequency variation of the proposed multi-order AAMs in the entire first Brillouin zone are calculated, and it was found that the dispersion relation of these AAMs can be obtained by scanning along the boundary of the simplest Brillouin zone. The sound transmission properties and effective medium parameters of the first-order to the third-order AAMs are systematically calculated and studied, which proves that the range of higher transmission loss occurs, and the frequency range of negative effective parameters and the bandgap range are consistent. The experimental results verify the effectiveness of the asymmetric design for broadband sound isolation and the correctness of the simulation calculation. This research shows that breaking the symmetry of the structure enables the acoustic metamaterial to exhibit a better broadband sound isolation performance, which provides new inspiration for further expanding the application field of asymmetric acoustic metamaterials.

## Figures and Tables

**Table 1:** Omnidirectional bandgaps of the AAMs with the square lattice.

AAMs	Frequency Ranges of Omnidirectional Bandgaps		Proportion of Bandgaps in the Subwavelength Range	
The first-order AAMs	BG-1	[0.1470, 0.2097]	6.27%	63.6%
	BG-2	[0.2668, 0.8401]	57.33%	
The second-order AAMs	BG-1	[0.1190, 0.2483]	12.93%	75.96%
	BG-2	[0.2949, 0.5990]	30.41%	
	BG-3	[0.6443, 0.7737]	12.94%	
	BG-4	[0.8032, 1]	19.68%	
The third-order AAMs	BG-1	[0.1134, 0.2732]	15.98%	76.84%
	BG-2	[0.3203, 0.5515]	23.12%	
	BG-3	[0.5927, 0.8217]	22.9%	
	BG-4	[0.8516, 1]	14.84%	

## Data Availability

The data used to support the findings of this study are included in this article.
